# Analysis of changes in bacterial diversity in healthy and bacterial wilt mulberry samples using metagenomic sequencing and culture-dependent approaches

**DOI:** 10.3389/fpls.2023.1206691

**Published:** 2023-08-23

**Authors:** Ting Yuan, Izhar Hyder Qazi, Jinhao Li, Peijia Yang, Hongyu Yang, Xueyin Zhang, Weili Liu, Jiping Liu

**Affiliations:** South China Agriculture University, College of Animal Science, Regional Sericulture Training Center for Asia-Pacific, Guangzhou, Guangdong, China

**Keywords:** bacterial wilt, drug-resistant bacteria, *Enterobacter cloacae* complex, *Klebsiella*, mulberry, opportunistic pathogens, RSSC

## Abstract

**Introduction:**

Mulberry bacterial wilt is a serious destructive soil-borne disease caused by a complex and diverse group of pathogenic bacteria. Given that the bacterial wilt has been reported to cause a serious damage to the yield and quality of mulberry, therefore, elucidation of its main pathogenic groups is essential in improving our understanding of this disease and for the development of its potential control measures.

**Methods:**

In this study, combined metagenomic sequencing and culture-dependent approaches were used to investigate the microbiome of healthy and bacterial wilt mulberry samples.

**Results:**

The results showed that the healthy samples had higher bacterial diversity compared to the diseased samples. Meanwhile, the proportion of opportunistic pathogenic and drug-resistant bacterial flora represented by *Acinetobacter* in the diseased samples was increased, while the proportion of beneficial bacterial flora represented by *Proteobacteria* was decreased. *Ralstonia solanacearum* species complex (RSSC), *Enterobacter cloacae* complex (ECC), *Klebsiella pneumoniae*, *K. quasipneumoniae*, *K. michiganensis*, *K. oxytoca*, and *P. ananatis* emerged as the main pathogens of the mulberry bacterial wilt.

**Discussion:**

In conclusion, this study provides a valuable reference for further focused research on the bacterial wilt of mulberry and other plants.

## Introduction

Mulberry is a perennial dicotyledonous tree or shrub (Dai et al., 2020) that is widely cultivated throughout subtropical and temperate regions, and has a significant economic value (Xie et al., 2020). Mulberry leaves are exclusively used as a food source for the domesticated silkworm *Bombyx mori* L. (Ji et al., 2008; Chan et al., 2016). Besides its use as silkworm forage, mulberry is now used as a raw material in animal feed (Jiang et al., 2022), medicine (Meng et al., 2020) and food industry (Maqsood et al., 2022). However, the occurrence of mulberry diseases has seriously affected the healthy and stable development of the sericulture industry (Dong et al., 2021). For instance, mulberry bacterial wilt is a destructive disease that seriously affects the yield and quality of mulberry (Dong et al., 2021). Mulberry bacterial wilt was first reported in 1969 in Shunde City, Guangdong province of China, and has spread to most mulberry planting areas in Guangdong (Lai et al., 1979). Mulberry bacterial wilt is still prevalent in the main sericulture-producing areas of Guangdong, Guangxi and other places in China (Dai et al., 2016), and has been reported in many other mulberry planting areas in the country.

Mulberry bacterial wilt is a vascular disease which is difficult to diagnose with the naked eye at the initial stage of infection. However, in the middle stage of the disease, the leaves lose moisture and then curl or wilt, turning black or brown. In the late stage of the disease, the leaves of the whole plant are withered until they fall off, the xylem turns brown streaked or dark brown, and white pus-like bacteria overflow from the cross-section of the diseased root (Wang et al., 2008; Zhu et al., 2010; Zhou et al., 2021; Luo et al., 2022; Yuan et al., 2023a).

The pathogen of mulberry bacterial wilt has complex and diverse characteristics. Lai et al. (1979) isolated and identified the pathogen of mulberry bacterial wilt for the first time. Initially, *Pseudomonas solanacearum* was considered as a pathogen causing mulberry bacterial wilt, which was later renamed as *Ralstonia solanacearum*, and now classified as *R. pseudosolanacearum*. Wang et al. (2008) reported for the first time that the mulberry wilt was also caused by *Enterobacter cloacae* complex (*ECC*). Subsequently, Zhu et al. (2010) isolated *E. mori* from mulberry wilt disease samples. Zhou et al. (2021) isolated *E. roggenkampii* strain KQ-01 from the bacterial wilt-resistant mulberry cultivar YS283, which can cause mulberry wilt. Luo et al. (2022) isolated *Klebsiella michiganensis* AKKL-001 from mulberry bacterial disease samples, which can also cause mulberry wilt. Recently, *Pantoea ananatis* strain LCFJ-001 was isolated from mulberry bacterial wilt disease samples and was reported to cause mulberry wilt (Yuan et al., 2023a).

Currently reported pathogens of mulberry bacterial wilt can be divided into four categories: *Ralstonia*, *Enterobacter*, *Klebsiella*, and *Pantoea* (**Supplementary Figure 1**). The gradual increase in sericulture production and exchange activities in the recent times has also led to an increased occurrence of bacterial wilt in mulberry fields in China, leading to significant challenges to the healthy development of the sericulture industry in the country. As this complex disease is caused by a number of pathogens, it still remains to be known which pathogen is the main pathogen, making its prevention and control difficult. It has been reported that the occurrence of plant diseases is related to changes in their crop microbiome, and that the study of changes in their microbiome can further reveal their pathogenesis (Li et al., 2022). Therefore, in order to further understand the basis of pathogenesis and provide a valuable reference for prevention and control, this study was carried out to explore the changes in mulberry microbiome in bacterial wilt and healthy samples of mulberry. In the present study, we collected (2017 to 2022) 35 mulberry bacterial wilt disease samples from Guangdong, Guangxi, and other regions of China. The diseased mulberry samples were isolated and tested for pathogenicity of pathogenic bacteria. At the same time, due to the limitations of traditional culture-dependent method, we also used the metagenomic sequencing to further explore the main pathogenic groups in the diseased and healthy mulberry samples.

## Materials and methods

### Metagenomic sequencing of mulberry samples

#### Collection of mulberry samples

A survey of mulberry fields where mulberry wilt was prevalent in Liucheng (109.24°, 24.65°) and Rong’an (109.35°, 25.15°) counties of Guangxi, China was conducted (see **Supplementary Figures 2A–I** for description). The mulberry samples with typical disease symptoms in the field were processed for laboratory verification.

#### Metagenomic sequencing of mulberry samples

The pH values of the diseased (wilted) plants and rhizosphere soil of typical mulberry in Liucheng and Rong’an were tested. Eight samples were collected (**Supplementary Table 1**) and sent to the Science Corporation of Gene Co., Ltd. for metagenomic sequencing to analyze the types, and abundance of pathogens in the samples.

#### Extraction of genomic DNA

The genome DNA was extracted from samples using the Ezup Column Bacteria genomic DNA purification kit (Sangon Biotech (Shanghai) Co., Ltd., China). DNA purity and concentration were measured by gel electrophoresis and *NanoDrop* 2000 (Thermo Scientific) spectrophotometer (Yuan et al., 2023b).

#### Amplification of the target region

The *16S rRNA gene* consists of nine hypervariable regions flanked by regions of more conserved sequence. To maximize the effective length of PE 250 sequencing reads of Illumina HiSeq2500, the region encompassing the V3 and V4 hypervariable regions of the *16S rRNA* gene was targeted for sequencing. The V3-V4 hypervariable region was amplified using a specific primer with the barcode (**Supplementary Table 2**). All PCRs were carried out in 40 μL reactions with 20 μL of 2×Taq MasterMix, 0.5 μM forwards and reverse primers, and approximately 10 ng of template DNA. Temperature cycling consisted of denaturation at 94°C for 2 min, followed by 30 cycles of denaturation at 94°C for 30 s, annealing at 60°C for 30 s, and elongation at 72°C for 20 s., and finally, 72°C for 7 min. The purity and concentration of all amplicons were characterized by gel electrophoresis and Qubit@ 2.0 Fluorometer (Thermo Scientific). The amplicons with bright main strips and the right length were chosen for the subsequent experiments (Yuan et al., 2023b).

#### Library preparation and metagenomic sequencing

PCR products with different barcodes were mixed in equidensity ratios. Then, a mixture of PCR products was used to prepare pair-end sequencing libraries. The libraries were generated following the Illumina HiSeq 2500 standard protocol (Illumina, Inc., San Diego, CA). Paired-end reads (250 bp) were generated on the Illumina HiSeq2500 platform. Three replicates of each sample were used for metagenomic sequencing (Yuan et al., 2023b). The metagenomic sequencing data have been uploaded to the NCBI (National Center for Biotechnology Information) with accession number PRJNA911049. Finally, based on taxonomy, the abundance of each bacterial genus was counted, and Origin 2019b software (OriginLab Corporation, Northampton, MA, USA) was used to make bacterial abundance maps of different samples and the Shannon, Chao-1 and Simpson values of each sample were calculated.

### Analysis of mulberry samples using culture-dependent approach

#### Collection of mulberry samples

During 2019 to 2022, a total of 35 samples of diseased plants were collected from Guangxi, Guangdong and Hainan in China (**Supplementary Table 3**). *M. atropurpurea* varieties Lun40 and Kangqing10 were used as the healthy group (**Supplementary Table 3**), and 20 copies of each variety were collected in mulberry field of the South China Agricultural University, Guangzhou, Guangdong, China (113.35°, 23.17°).

#### Isolation of bacteria from mulberry samples

The experimental design is depicted in **Supplementary Figure 3**. Firstly, the collected diseased or healthy roots were rinsed under the faucet, and the surface stains were washed with soapy water and the samples were wiped with a clean gauze. The roots were cut into small sections of three centimeters in length using clean scissors. Then, the sections were soaked in 75% ethanol for 1 min, rinsed with sterile water three times, soaked in 0.1% mercuric chloride for 5 min, and rinsed with sterile water five times. Then, the surface-sterilized small section was placed in a sterile glass petri dish, the xylem in the center was removed with sterile tweezers and scissors, cut into pieces and ground in a sterile mortar. The ground xylem was placed in 10 mL of sterile saline and in a shaker at 28°C and 140 r/min for 10 minutes to form a liquid containing xylem bacteria. The liquid was then removed and diluted eight times according to the 10-fold dilution method (Yuan et al., 2023a).

A total of 0.1 mL of each gradient was spread evenly on Lysogeny Borth (LB) agar plates (Guangdong Huankai Co., Ltd., China) and nutrient agar plates (Guangdong Huankai Co., Ltd., China). Then, the plates were placed in a biochemical incubator at 28°C for two days for cultivation. Finally, single colonies were picked from LB agar and nutrient agar media and drawn on new nutrient agar plates, and each colony was purified for seven generations (Yuan et al., 2023a).

### Classification of bacteria

Classification of the bacteria was based on analysis of *16S rRNA gene* using universal primer 27F/1492R (Bredow et al., 2015). All strains were inoculated in nutrient broth medium (Guangdong Huan Kai Co., Ltd., China) and placed in a shaker at 28°C and 140 r/min for 12 h. Bacterial genomic DNA was extracted using the Ezup Column Bacteria Genomic DNA purification kit (Sangon Biotech (Shanghai) Co., Ltd., China). DNA from all purified isolates was used for PCR amplification of the *16S rRNA gene*, which was performed in a 25 μL volume under the following conditions: one cycle of 98°C for 4 min, followed by 30 cycles of 98°C for 30 seconds, 55°C for 30 seconds, 72°C for 1 minute, and final an extension at 72°C for 10 minutes. The PCR-amplified products were transferred to a laboratory in Shanghai, China, at Sangon Biotechnology Co. Ltd. in Shanghai, China, and then sequenced by the Sanger method (Yuan et al., 2023a).

The generated sequences were aligned using BioEdit software version 7.0 and then subjected to analysis by the Basic Local Alignment Search Tool (BLAST) search program of the NCBI (https://blast.ncbi.nlm.nih.gov/Blast.cgi) to determine the sequence homology with closely related organisms (Altschul et al., 1997). Microorganisms with high homology (97-100%) were selected as the closest matches. All bacterial isolates were assigned to the genus level separately based on information from the closest microorganisms. In addition, the NCBI taxonomic database was used to classify all bacterial strains at the phylum, class, order, and family levels (Yuan et al., 2023b). All bacterial *16S rDNA* sequences generated in this study have been submitted to the NCBI. The accession numbers OP990608-OP990981 are bacterial *16S rDNA* sequences derived from healthy samples; OP989957-OP990607 are bacterial *16S rDNA* sequences derived from diseased samples. Finally, based on the *16S rRNA gene* bacterial identification results, the abundance of each bacterial genus was counted, and the bacterial abundance maps of different samples were made using Origin 2019b software (OriginLab Corporation, Northampton, MA, USA) and the Shannon, Chao-1, and Simpson values of healthy and diseased samples were calculated.

### *16S rRNA gene* phylogenetic tree construction

The *16S rDNA* sequences of typical strains of *Ralstonia*, *Enterobacter*, *Klebsiella*, and *Pantoea* were downloaded from the List of Prokaryotic names with Standing in Nomenclature (https://lpsn.dsmz.de/). The *16S rDNA* of six strains of *Ralstonia*, 30 strains of *Enterobacter*, 12 strains of *Klebsiella*, and 12 strains of *Pantoea* were selected for construction of phylogenetic trees (**Supplementary Table 4**). At the same time, the *16S rDNA* sequences of strains identified in our laboratory (**Supplementary Table 4**) and the *16S rDNA* sequences downloaded from the NCBI were used as references. The sequences were compared using MUSCLEv.3.8.31 software. The phylogenetic trees were constructed using the maximum likelihood tree with MEGA-X software, and the bootstrap value was set to 1000 (Yuan et al., 2023a).

### Cultivation of mulberry branches

Healthy 15-year-old healthy *M. atropurpurea* cultivar Lun40 (susceptible to bacterial wilt) obtained from the South China Agricultural University mulberry field (Guangzhou, Guangdong, China), (113.35°, 23.17°) was selected as plant material for this study. The samples were collected in December 2021. Firstly, old branches of Lun40 with a diameter of 0.5-0.75 centimeter were selected and cut into stem segments of 10-12 centimeters in length and containing three lateral shoots. The stems were washed with soapy water to remove surface dust and soaked in 0.5% sodium hypochlorite solution for five hours. The stems were then inserted into the sterile MS liquid medium and placed in an artificial climate incubator at 25°C, 12 h/d light, and 85% humidity. The culture was incubated for 25 days until the lateral shoots sprouted and exhibited 2-3 leaves. During this period, the sterile MS liquid medium was changed every day (Yuan et al., 2023b).

### Pathogenicity test

To investigate the pathogenicity of *Ralstonia*, *Enterobacter*, *Klebsiella*, and *Pantoea*, the following procedures were adopted: 1) the pure cultures of all the bacteria (**Supplementary Table 4**) were placed in nutrient broth medium overnight. The overnight cultured bacteria solution was adjusted to OD600 nm=0.1 with sterile MS liquid medium. 2) After cultivation, the Lun40 mulberry branch with 2~3 leaves was placed into the diluted bacterial solution. The sterile MS liquid medium was set as the healthy group. 3) The treated Lun40 mulberry branches were cultured for 12 days in an artificial climate incubator at 28°C, 12 h/d light, and 85% humidity, and the disease incidence in plants was observed. 4) Morbidity rate= (A-B)/C×100%. A: The total number of diseased mulberry branches in the experimental group; B: The total number of diseased mulberry branches in the control group; C: The total number of mulberry branches (Yuan et al., 2023b).

Analysis of variance (ANOVA) was performed using Excel software. Each set of experiments for each pathogen species was tested using six healthy mulberry branches. With the sterile MS liquid medium as a control, each group had three replicates (Yuan et al., 2023b).

### Data statistics

Data analysis was performed using a one-way analysis of variance (Levene’s test was used to evaluate the equality of variance before analysis), and the least significant difference test was used to determine the significant difference between the means as a *post hoc* analysis. *P*<0.05 was considered significant. Excel 2016 software (Microsoft, Redmond, WA, USA) and Origin 2019b 64Bit were used to analyze and map the data.

## Results

### Analysis of metagenomic sequencing quality

The description of sequencing data of the bacterial *16S rDNA* V3-V4 regions collected from mulberry xylem and rhizosphere soil are shown in **Table 1**. Briefly, the GC was greater than 53%, Q20 was greater than 96%, and Q30 was greater than 94%. This indicates a low sequencing error rate and high quality and reliability of the data.

**Table 1:** Description of metagenomic sequencing data.

Sample	Reads(#)	Base(nt)	GC(%)	Q20(%)	Q30(%)
QKB04^**^	167,870	41,967,500	53.74	96.60;88.78	94.03;82.91
QKB06^*^	183,782	45,945,500	54.6	96.82;89.16	94.30;83.23
QKB08^**^	167,210	41,802,500	54.68	96.81;89.51	94.33;83.81
QKB10^*^	188,724	47,181,000	54.04	96.81;89.58	94.36;83.96
QKB03^**^	156,488	39,122,000	55.03	96.72;89.52	94.18;83.93
QKB05^*^	120,666	30,166,500	54.12	96.81;89.74	94.39;84.16
QKB07^**^	125,124	31,281,000	53.49	96.80;88.99	94.32;83.20
QKB09^*^	136,356	34,089,000	53.73	96.90;89.79	94.53;84.26

“*”: healthy group; “**” diseased group; Reads(#): The total number of reads for sequencing; Bases (nt): the number of bases for sequencing = the total number of reads for sequencing×150 (150 is the length of the sequencing read); Q20 (%): the proportion of bases with a sequencing quality value greater than 20 (error rate less than 1%) in R1 and R2 sequencing reads; Q30 (%): the proportion of bases with sequencing quality more significant than 30 (error rate less than 0.1%) in R1 and R2 sequencing reads; GC (%): GC proportion.

### Analysis of diversity of bacterial community in mulberries based on metagenomic sequencing

As shown in **Table 2**, the bacterial community OTU numbers, Shannon index, Chao-1 index and Simpson index of the diseased rhizosphere soil (QKB04 and QKB08) and xylem (QKB03 and QKB07) were lower compared to the healthy rhizosphere soil (KB06 and QKB10) and xylem (QKB05 and QKB09). From these, the bacterial diversity of the rhizosphere soil and xylem of the diseased mulberry was lower compared to the healthy mulberry.

**Table 2:** The number of OTUs and diversity index of the read sequence (Tags) bacterial community of the sequenced branch samples.

Sample	Shannon	Chao-1	Simpson	OTU	Tags
QKB04^**^	6.90^c^	5554.90^b^	0.87^b^	1364^b^	5676^a^
QKB06^*^	7.39^d^	15270.62^e^	0.93^c^	5543^f^	30833^c^
QKB08^**^	6.77^c^	12231.04^d^	0.89^b^	4329^e^	26340^b^
QKB10^*^	7.42^d^	15988.35^e^	0.94^c^	6531^g^	39377^d^
QKB03^**^	5.36^a^	6968.12^b^	0.81^a^	2750^c^	24642^b^
QKB05^*^	5.84^b^	7704.39^c^	0.90^bc^	2860^c^	27899^b^
QKB07^**^	5.48^a^	3178.83^a^	0.88^b^	862^a^	5387^a^
QKB09^*^	5.23^a^	6928.89^b^	0.88^b^	3303^d^	39219^c^

“*”: indicates the healthy group; “**” indicates the diseased group. Different superscript letters in the vertical column indicate significant differences between means by one-way analysis of variance (ANOVA) and least significant difference (LSD) test (P<0.05).

### Analysis of bacterial community composition of mulberries based on metagenomic sequencing

**Figure 1:**
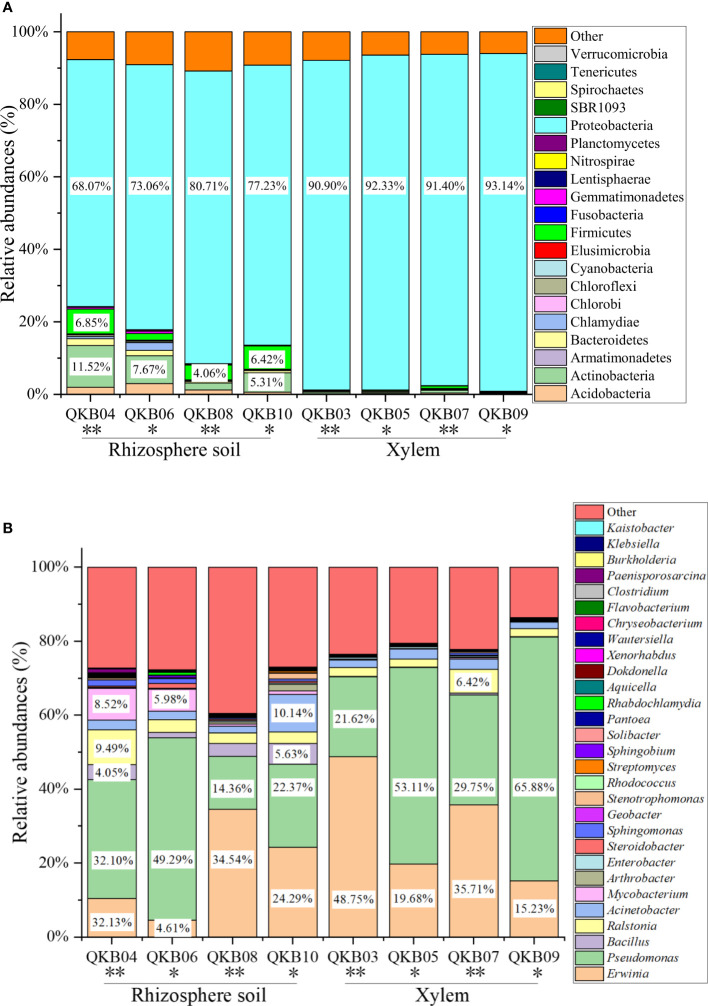
Abundance distribution of bacterial populations based on metagenomic sequencing at the phylum **(A)** and genus **(B)** levels. QKB04: Liucheng (109.24°, 24.65°) diseased sample rhizosphere soil; QKB06: Liucheng (109.24°, 24.65°) healthy sample rhizosphere soil; QKB08: Rong’an (109.35°, 25.15°) diseased sample rhizosphere soil; QKB10: Rong’an (109.35°, 25.15°) healthy sample rhizosphere soil; QKB03: Liucheng (109.24°, 24.65°) diseased sample xylem; QKB05: Liucheng (109.24°, 24.65°) healthy sample xylem; QKB07: Rong’an (109.35°, 25.15°) diseased sample xylem; QKB09: Rong’an (109.35°, 25.15°) diseased sample xylem; “*”: indicates the healthy group; “**” indicates the diseased group.

The phylum-level abundance distribution of bacterial populations in the mulberry rhizosphere soil was associated with 17 phyla (**Figure 1A**). From these, Proteobacteria had the highest abundance in diseased and healthy samples, followed by Actinomycetes and unclassified bacteria. At the genus level, the taxonomic sequence of the mulberry rhizosphere soil was associated with 49 genera (**Figure 1B**). The abundance distribution of the dominant flora are shown in **Table 3**. *Pseudomonas* accounted for the largest proportion, followed by *Mycobacteria*, *Erwinia* and *Ralstonia*, respectively. The *Pseudomonas* disease samples showed a significant downward trend (*P*<0.05), whereas the *Erwinia* disease samples showed a significant upward trend (*P*<0.05). Interestingly, there was no significant difference in the abundance of *Ralstonia* between healthy and diseased samples (P<0.05).

**Table 3:** Richness of important bacterial genera in different samples based on metagenomic sequencing data.

Species	QKB04^**^	QKB06^*^	QKB08^**^	QKB10^*^	QKB03^**^	QKB05^*^	QKB07^**^	QKB09^*^
*Acinetobacter*	2.66%	2.15%	1.78%	10.15%	2.05%	2.73%	2.88%	1.73%
*Arthrobacter*	0.15%	0.14%	0.55%	1.79%	0.075%	0.045%	0.11%	0.018%
*Bacillus*	4.05%	1.38%	3.38%	5.63%	0.14%	0.16%	0.43%	0.13%
*Enterobacter*	0.12%	0.12%	0.43%	0.45%	0.42%	0.44%	0.37%	0.19%
*Erwinia*	10.37%	4.62%	34.54%	24.30%	48.76%	19.69%	35.71%	15.23%
*Klebsiella*	0.017%	0.15%	0.030%	0.086%	0.040%	0.097%	0.019%	0.14%
*Mycobacterium*	8.52%	5.98%	0.68%	0.90%	0.19%	0.12%	0.32%	0.11%
*Paenisporosarcina*	1.12%	0.019%	0.042%	0.053%	0.0035%	0.016%	0.037%	0.0025%
*Pantoea*	0.14%	0.039%	0.072%	0.070%	0.11%	0.15%	0%	0.079%
*Pseudomonas*	32.13%	49.29%	14.37%	22.37%	21.62%	53.11%	29.76%	65.88%
*Ralstonia*	9.49%	3.54%	2.86%	3.17%	2.34%	2.15%	6.42%	2.15%
*Sphingomonas*	1.71%	1.27%	0.28%	0.51%	0.15%	0.21%	0.59%	0.12%
*Stenotrophomonas*	0.45%	0.15%	0.11%	1.53%	0.13%	0.11%	0.30%	0.13%
*Steroidobacter*	0.29%	1.34%	0.36%	0.48%	0.11%	0.10%	0.22%	0.090%

“*”: indicates the Healthy group; “**” indicates the Diseased group.

The phylum-level abundance distribution of bacterial populations in mulberry xylem was associated with seven phyla (**Figure 1A**). Proteus was the first dominant bacterial group in both healthy and diseased mulberry samples and its abundance accounted for more than 90% in both healthy and diseased groups. Many sequences in xylem could not be classified (7.8% richness), indicating the diversity of the xylem bacteria. At the genus level, the bacteria in the mulberry xylem part were related to 23 genera (**Figure 1B**). The abundance distribution of the dominant bacterial taxa is shown in **Table 3**. *Pseudomonas* was found to be the most abundant, followed by *Erwinia* and *Ralstonia*, respectively. Interestingly, the abundance of *Pseudomonas* was lower in the diseased group compared to the healthy mulberry group, whereas abundance of *Erwinia* and *Ralstonia* showed a reverse trend.

### Bacterial composition and diversity in mulberries based on a culture-dependent approach

A total of 1052 strains of the xylem bacteria were isolated from all samples. From these, 389 strains were from the healthy mulberry samples (CKS) (**Supplementary Table 5**), 663 strains were from the diseased (bacterial wilt) mulberry samples (MBWS) (**Supplementary Table 6**). Based on the results of *16S rRNA* gene, CKS culturable strains were divided into 58 genera, distributed in 4 phyla, 6 classes, 18 orders and 28 families (**Supplementary Table 5**). The Shannon, Simpson and Pielou values were 3.03, 0.90 and 0.75, respectively (**Table 4**). The culturable strains of MBWS were divided into 69 genera, distributed in 4 phyla, 9 classes, 17 orders and 31 families (**Supplementary Table 6**). The values of Shannon, Simpson and Pielou were 3.17, 0.92 and 0.75, respectively (**Table 4**). This finding indicated that the diversity of the xylem bacteria in the MBWS samples was slightly higher compared to the CKS samples (*P*>0.05).

**Table 4:** Profiles of bacterial community diversity in the biomass of diseased and healthy mulberry samples based on culture-dependent approach.

	*M. atropurpurea*	
	MBWS**	CKS*
Number of isolates	663^b^	389^a^
Number of genera	69^b^	58^a^
Shannon-Weaver (H’)	3.17^a^	3.03^a^
Simpson’s index (D)	0.92^a^	0.90^a^
Pielou’s evenness (E)	0.75^a^	0.75^a^

“*”Healthy group; “**” Diseased group; MBWS: mulberry bacterial wilt sample. CKS: Healthy samples (healthy mulberry samples). Different letters in the same row indicate significant difference between means by one-way analysis of variance (ANOVA) and least significant difference (LSD) test (P<0.05).

**Figure 2:**
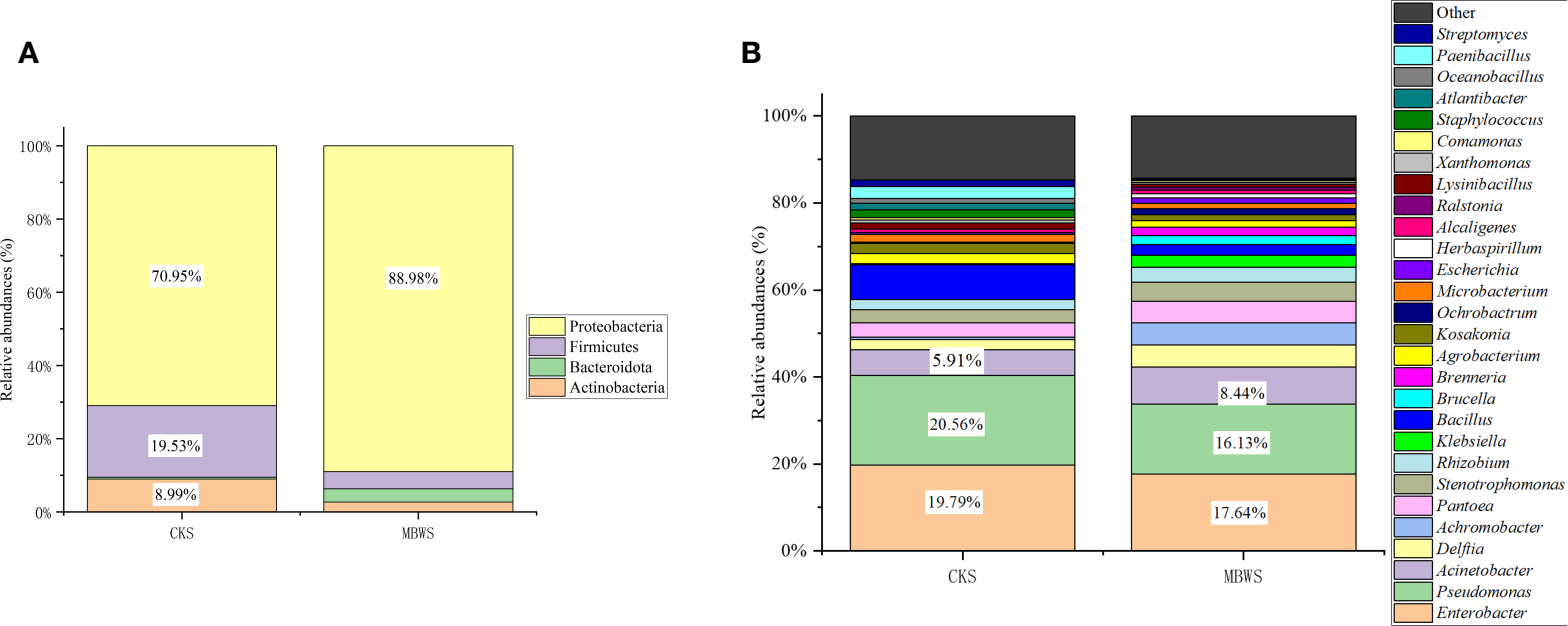
Relative abundance (%) of cultivable bacteria in different communities isolated from healthy and diseased mulberry at the phylum **(A)** and genus **(B)** levels. MBWS, mulberry bacterial wilt sample; CKS, Healthy samples (healthy mulberry samples).

All isolates belonged to Actinobacteria, Bacteroidetes, Firmicutes and Proteobacteria phyla. From these, Proteobacteria was found to the dominant phylum in the bacterial community of mulberry xylem (**Figure 2A**). The most abundant Proteobacteria (CKS 70.95%, MBWS 88.98%) mainly contained the Alphaproteobacteria, Betaproteobacteria, and Gammaproteobacteria-like bacteria. Firmicutes was the second most dominant phylum (CKS 19.53%, MBWS 4.67%) and contained only bacilli, consisting of *Bacillus* (**Figure 2A**). *Actinomycetes* was the third most dominant bacterial phylum (CKS 8.99%, MBWS 2.71%) and was represented by *Microbacteria* (**Figure 2A**). At the genus level (**Figure 2B**), *Pseudomonas*, *Enterobacter*, and *Acinetobacter* were found to be the main groups of the xylem bacteria.

### Analysis of bacterial community of mulberries based on culture-dependent approach

**Figure 3:**
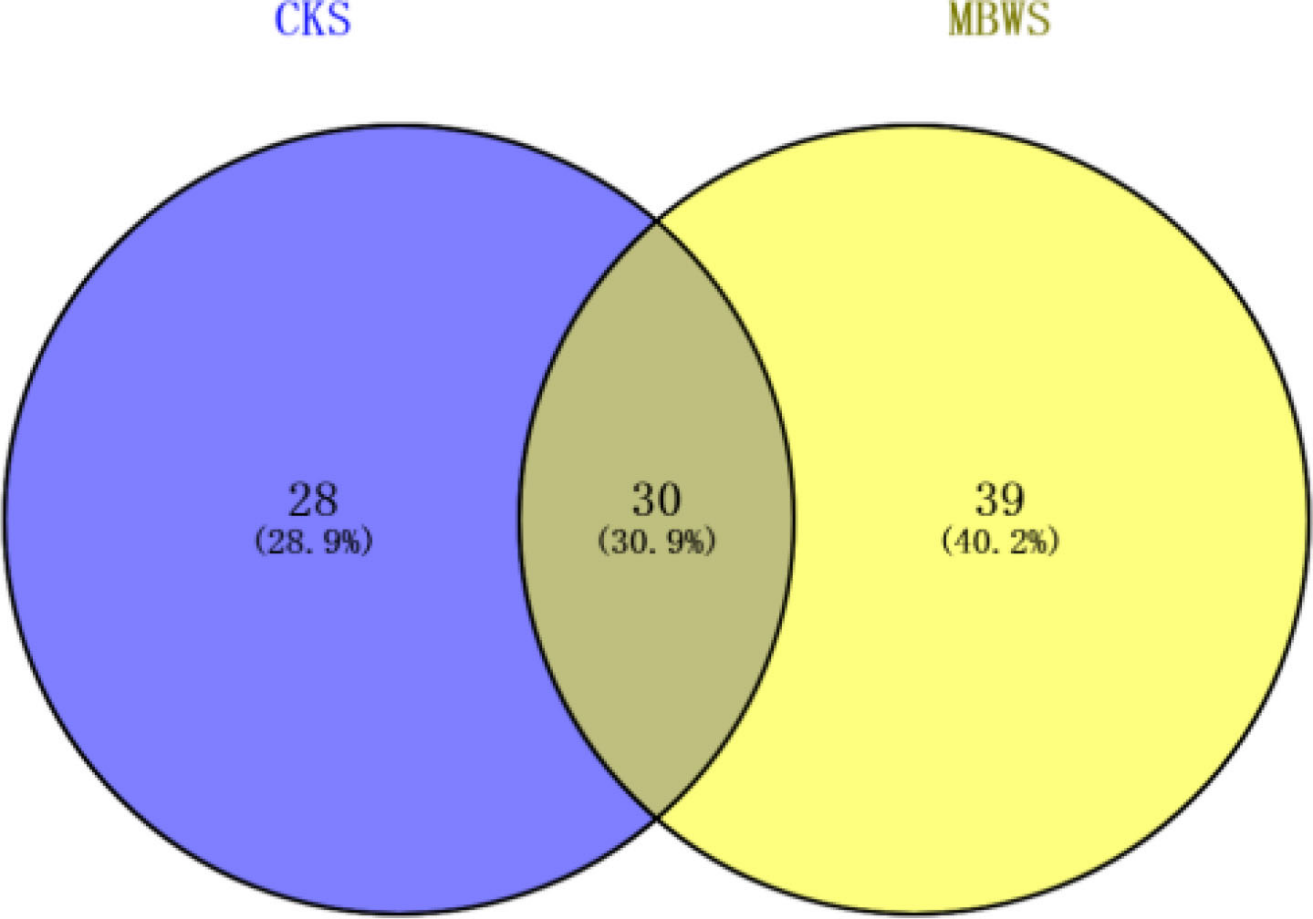
Bacterial Venn diagrams of healthy and diseased mulberry samples. MBWS, mulberry bacterial wilt sample; CKS, healthy mulberry samples.

Unique and shared bacterial genera between healthy and diseased mulberry groups are shown in the Venn diagram (**Figure 3**). The number of shared attributes across all groupings was 33 (**Figure 3**). In addition, the number of unique genera in the MBWS was higher than the number of unique and shared genera in the CKS group. Genera including *Enterobacter*, *Pseudomonas*, *Acinetobacter*, *Delftia*, *Pantoea*, *Stenotrophomonas*, *Rhizobium*, *Bacillus*, *Agrobacterium*, *Kosakonia*, and *Microbacterium*, with an average segregation rate of >1% in MBWS and CKS, were the 11 core genera of mulberry xylem bacteria (**Figure 2B**).

**Figure 4:**
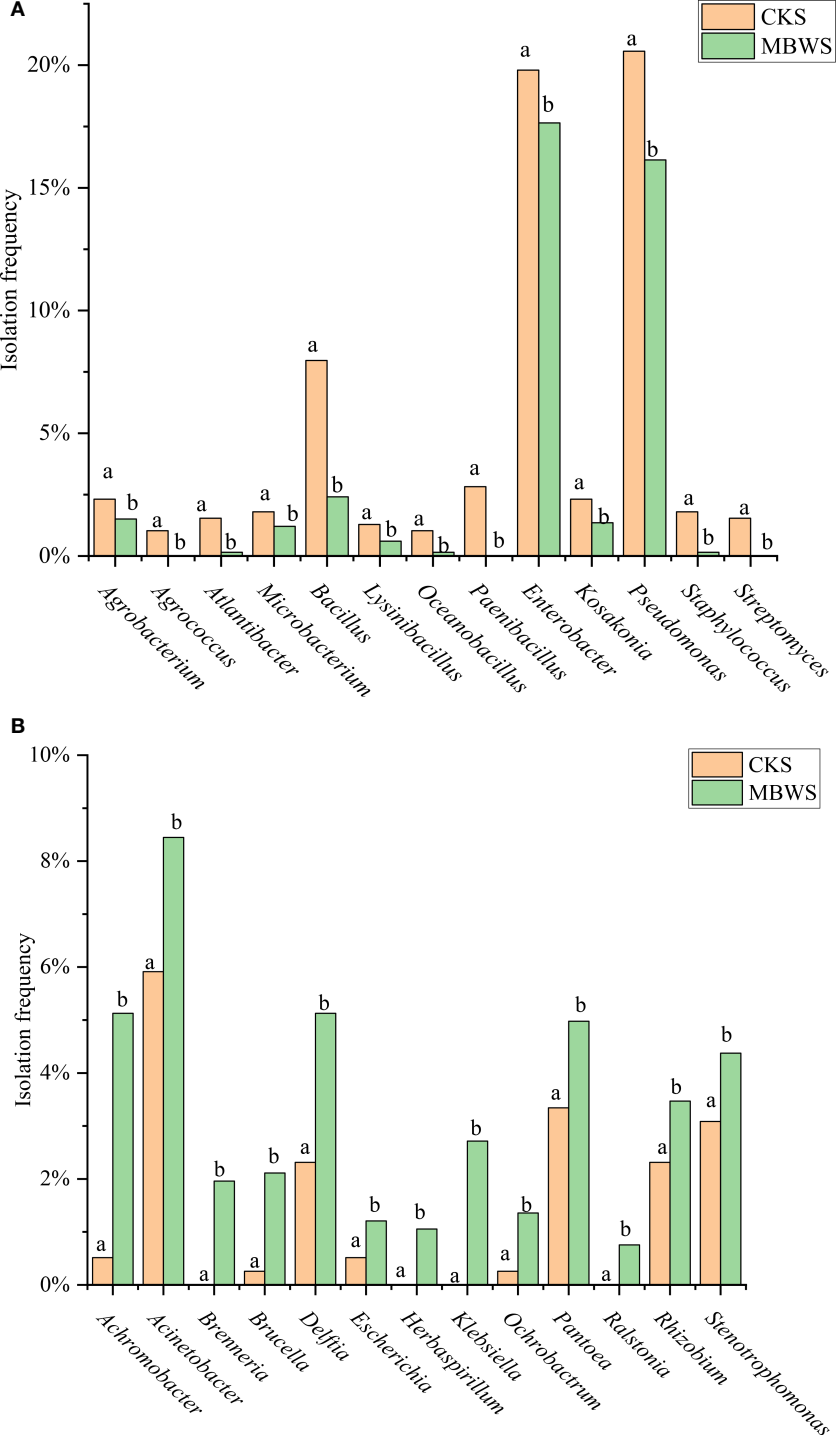
Isolation rates of abundant bacteria isolated from healthy and diseased mulberry. **(A)** Bacterial species and classification rates greater than CK in MBWS; **(B)** Bacterial species and classification rates greater than MBWS in CK; MBWS: mulberry bacterial wilt sample. CKS: Healthy samples (healthy mulberry samples). Bars with different letters indicate a significant difference between means by one-way analysis of variance (ANOVA) and least significant difference (LSD) tests (*P*< 0.05).

The separation frequencies of *Achromobacter*, *Acinetobacter*, *Brenneria*, *Brucella*, *Delftia*, *Escherichia*, *Herbaspirillum*, *Klebsiella*, *Ochrobactrum*, *Pantoea*, *Ralstonia*, *Rhizobium* and *Stenotrophomonas* in the CKS were significantly lower (P<0.05) compared to the MBWS group. Additionally, *Herbaspirillum*, *Brenneria*, *Klebsiella* and *Ralstonia* were isolated only in the diseased samples (**Figure 4A**).

The isolation frequencies of *Agrobacterium*, *Agrococcus*, *Atlantibacter*, *Microbacterium*, *Bacillus*, *Lysinibacillus*, *Oceanobacillus*, *Paenibacillus*, *Enterobacter*, *Kosakonia*, *Pseudomonas*, *Staphylococcus* and *Streptomyces* were significantly higher (P<0.05) in the CKS compared to the MBWS group. Additionally, *Agrococcus*, *Paenibacillus*, and *Streptomyces* were not isolated in the MBWS group (**Figure 4B**).

### The distribution of four main types of pathogenic bacteria in mulberries

**Figure 5:**
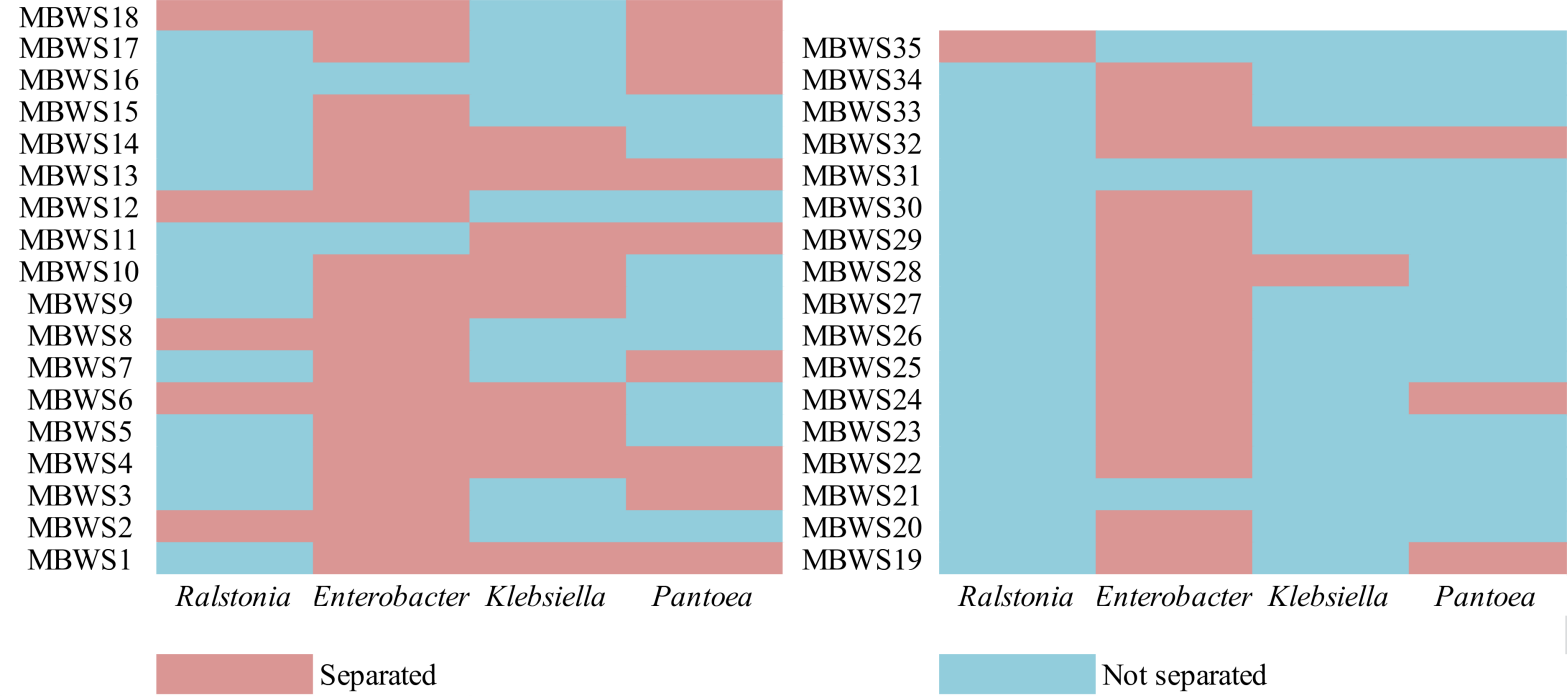
Isolation of *Ralstonia*, *Enterobacter*, *Klebsiella*, and *Pantoea* in different mulberry bacterial wilt samples.

In order to explore the main group of pathogenic bacteria causing bacterial wilt of mulberry, the distribution of *Ralstonia*, *Enterobacter*, *Klebsiella*, and *Pantoea* in 35 diseased samples was analyzed (**Figure 5**). From these diseased samples, *Ralstonia*, *Enterobacter*, *Klebsiella*, and *Pantoea* were isolated from 6 (17.14%), 30 (85.71%), 12 (34.28%) and 12 (34.28%) diseased samples, respectively. From the 30 diseased samples in which *Enterobacter* was isolated, *Klebsiella*, *Pantoea*, and *Ralstonia* were isolated from 10, 9, and 5 samples, respectively.

### Phylogenetic analysis of four main types of pathogenic bacteria

**Figure 6:**
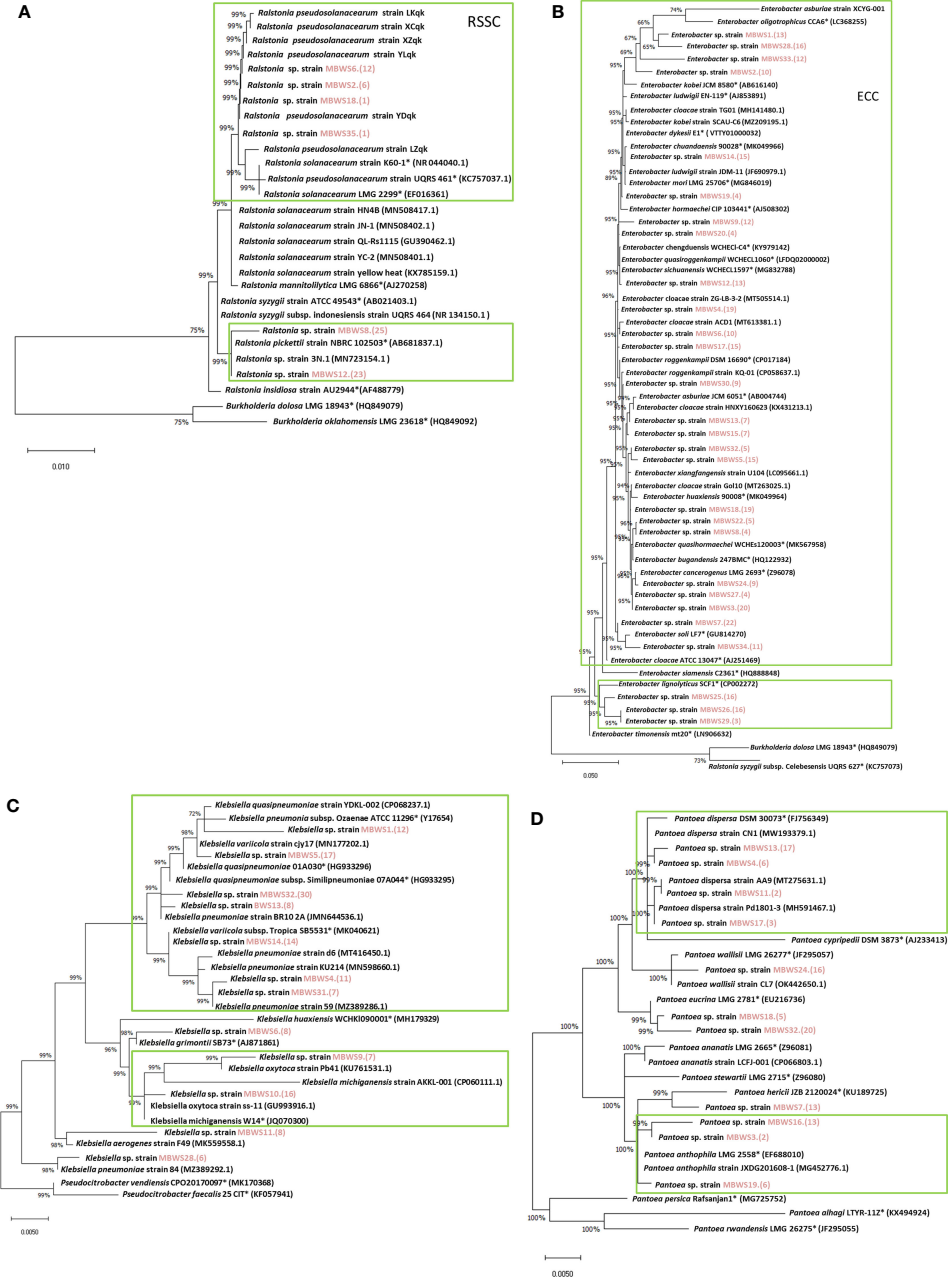
Phylogenetic trees of *Ralstonia* **(A)**, *Enterobacter* **(B)**, *Klebsiella* **(C)**, and *Pantoea* **(D)** based on *16S rRNA* genes. “*” indicates the representative species; the red marks are the isolates of this study.

Classification was based on *16S rDNA* sequences of *Ralstonia*, *Enterobacter*, *Klebsiella*, and *Pantoea* (**Figure 6**). *Ralstonia* was mainly concentrated in the RSSC and *R. pickettii* (**Figure 6A**). There were two main groups of *Enterobacter*: the ECC (*E. kobei*, *E. chengduensis*, *E. chuandaensis*, *E. hormaechei*, *E. cloacae*, *E. sichuanensis*, *E. roggenkampii*, E. *ludwigii*, and *E. cancerogenerus*.) and *E. lignolyticu*s (**Figure 6B**)*. Klebsiella* species were mainly divided into *K. michiganensis* and *K. oxytoca* (**Figure 6C**). *Pantoea* species were mainly clustered into two groups i.e., *P. dispersa* and *P. anthophila* (**Figure 6D**).

### Pathogenicity test of four main types of pathogenic bacteria

To further understand the role of *Ralstonia*, *Enterobacter*, *Klebsiella* and *Pantoea* in mulberry wilt, the pathogenicity test was conducted. As shown in **Table 5**, it was found that the average pathogenicity rate of *Ralstonia* derived from the MBWS was found to be 60.13%. The pathogenicity rate of *Ralstonia* with *16S rRNA* accumulated in the RSSC (*R. solanacearum* species complex) clade was higher than 43.33%, while the pathogenicity rate of *Ralstonia* aggregated in *R. pickettii* was 0% (**Figure 6A**). The average pathogenicity rate of *Enterobacter* derived from the MBWS was found to be 44.89%. From these, the main pathogenic group was concentrated in the ECC (*E. cloacae complex*) (**Figure 6B**), with greatly varying pathogenicity rates between them. The average pathogenicity rate of *Klebsiella* derived from the MBWS was found to be 44.76%. From these, the main pathogenic groups were *K. michiganensis*, *K. quasipneumoniae*, *K. oxytoca* and *K. pneumoniae* (**Figure 6C**), with greatly varying pathogenicity rates between them. The average pathogenicity rate of *Pantoea* derived from the MBWS was found to be 6.79%. From these, *P. ananatis* strain LCFJ-001 had the highest pathogenicity rate of 38.33%, while the others showed 0% pathogenicity rate (**Figure 6D**).

**Table 5:** Pathogenicity tests of *Ralstonia*, *Enterobacter*, *Klebsiella*, and *Pantoea*.

Ralstonia		Enterobacter		Klebsiella		Pantoea	
Strain Name	Morbidity %	Strain Name	Morbidity %	Strain Name	Morbidity %	Strain Name	Morbidity %
LKqk	100±0	XCYG-001	36.66±1.66	AKKL-001	85±2.88	LCFJ-001	38.33±1.66
LZqk	63.33±1.67	KQ-01	100±0	YDKL-002	55±2.88	MBWS1.(11)	0±0
XCqk	100±0	MBWS1.(13)	38.33±1.67	MBWS1.(12)	61.66±1.66	MBWS3.(2)	33.33±1.66
XZqk	100±0	MBWS2.(10)	83.33±1.67	MBWS4.(11)	41.67±1.67	MBWS4.(6)	0±0
YDqk	63.33±1.67	MBWS3.(20)	71.67±1.67	MBWS5.(17)	36.67±3.33	MBWS7.(13)	0±0
YLqk	43.33±1.67	MBWS4.(19)	71.67±1.67	MBWS6.(8)	0±0	MBWS11.(2)	0±0
MBWS2.(6)	45±2.88	MBWS5.(15)	36.67±1.67	MBWS9.(7)	70±2.88	MBWS13.(17)	0±0
MBWS6.(12)	0±0	MBWS6.(10)	100±0	MBWS10.(16)	41.66±1.66	MBWS16.(13)	0±0
MBWS8.(25)	0±0	MBWS7.(22)	16.67±1.67	MBWS11.(8)	0±0	MBWS17.(3)	0±0
MBWS12.(23)	63.33±1.67	MBWS8.(4)	0±0	MBWS13.(8)	61.67±1.6	MBWS18.(5)	0±0
MBWS18.(1)	80±2.88	MBWS9.(12)	36.67±1.67	MBWS14.(14)	70±2.88	MBWS19.(6)	16.66±1.66
MBWS35.(1)	63.33±1.67	MBWS10.(15)	68.33±4.41	MBWS28.(6)	0±0	MBWS24.(16)	0±0
		MBWS12.(13)	0±0	MBWS31.(7)	58.33±1.67	MBWS32.(20)	0±0
		MBWS13.(7)	73.33±1.67	MBWS32.(30)	45±2.88		
		MBWS14.(15)	36.67±1.67				
		MBWS15.(7)	0±0				
		MBWS17.(15)	35±2.88				
		MBWS18.(19)	55±2.88				
		MBWS19.(4)	0±0				
		MBWS20.(4)	53.33±1.67				
		MBWS22.(5)	0±0				
		MBWS23.(1)	100±0				
		MBWS24.(9)	51.67±1.67				
		MBWS25.(16)	0±0				
		MBWS26.(16)	0±0				
		MBWS27.(4)	100±0				
		MBWS28.(16)	100±0				
		MBWS29.(3)	0±0				
		MBWS30.(9)	100±0				
		MBWS32.(5)	16.67±1.67				
		MBWS33.(12)	55±2.88				
		MBWS34.(11)	0±0				
Morbidity mean	60.13%^a^	Morbidity mean	44.89%^b^	Morbidity mean	44.76%^b^	Morbidity mean	6.79%^c^

Different letters in the same row indicate significant difference between means by one-way analysis of variance (ANOVA) and least significant difference (LSD) test (P<0.05). Values represent the mean. Error bars indicate ± standard deviation.

## Discussion

Plant bacterial wilt is generally considered a highly destructive xylem disease caused by the *R. solanacearum* complex (RSSC). However, the advancement of bacterial wilt research shows that, in addition to other pathogens, the *E. cloacae* complex (ECC) can also cause bacterial wilt in African marigoldx (Jeevan et al., 2022), ginger (Cosmas et al., 2016) and mulberry plants (Wang et al., 2008; Zhu et al., 2011). Although the pathogenic bacteria of mulberry bacterial wilt are said to be complex and diverse, they mainly include *Ralstonia* (Pan et al., 2013), *Enterobacter* (Wang et al., 2008; Wang et al., 2010; Zhu et al., 2010; Zhu et al., 2011; Zhou et al., 2021), *Klebsiella* (Luo et al., 2022) and *Pantoea* (Yuan et al., 2023a). In order to better elucidate the interaction between the microbiome and mulberry, we used combined metagenomic sequencing and a culture-dependent approaches to explore the composition and diversity of bacterial communities in mulberry bacterial wilt samples.

We found 19 phyla and 112 genera in the diseased and healthy mulberry rhizosphere soil and xylem using Illumina HiSeq2500 sequencing. In contrast, four phyla and 97 genera were isolated and characterized using a culture-dependent approach. This discrepancy in the result infers that this phenomenon maybe linked to the inherent limitation of the culture-dependent method, as it is not entirely possible to isolate all xylem bacteria due to the limitation of the medium. On the contrary, it has been said that the metagenomic sequencing method can compensate for this limitation of the culture-dependent method (Zhang et al., 2020). Based on the results of metagenomic sequencing and Shannon, Chao-1, Simpson, and OTU, it was observed that the number and diversity of microbial flora in rhizosphere soil and xylem of healthy mulberry were higher than those of the diseased mulberry samples. Suhaimi et al. (2017) and Tao et al. (2022) also found that the diseased samples had a lower microbial diversity compared to the healthy samples. However, Kaushal et al. (2020) found contrasting result and reported higher OTU richness and diversity in the symptomatic roots. It is generally believed that a low microbial diversity in microbial communities favors pathogen invasion (Locey and Lennon, 2016). This argument is supported by finding of our recent report in which we found that the diversity of endophytes in highly resistant or moderately resistant varieties of mulberry bacterial wilt was significantly higher compared to the weakly resistant or susceptible varieties (Yuan et al., 2023b). This evidence also supports finding of the present study and demonstrate a potential link between diversity of microbial species and susceptibility.

Interestingly, both metagenomic sequencing and culture-dependent approaches revealed that Proteobacteria was a main phylum in both diseased and healthy mulberry rhizosphere soils and xylem, followed by Firmicutes and Actinobacteria. Proteobacteria, Firmicutes, and Actinobacteria were essential components of bacteria in healthy and diseased mulberry xylem. This finding is in line with the evidence reported by Yuan et al. (2023b); Xu et al. (2019) and Ou et al. (2019). The culture-dependent method also revealed that Proteobacteria and Bacteroidetes had greater (*P*<0.05) abundance in the diseased mulberry xylem compared to the healthy samples. In contrast, Actinobacteria and Firmicutes had greater (*P*<0.05) abundance in the healthy xylem compared to the diseased samples. Interestingly, Kaushal et al. (2020) have reported that the abundance of Proteobacteria and Actinobacteria showed a similar trend in the banana Mchare cultivar. Suhaimi et al. (2017) have also reported that the healthy samples had higher richness of Proteobacteria than the diseased samples.

At the subordination level, metagenomic sequencing revealed that *Erwinia*, *Pseudomonas*, *Ralstonia*, and *Acinetobacter* were the dominant genera, accounting for more than 1% of the eight samples tested. On the other hand, the culture-dependent approach revealed that *Enterobacter*, *Pseudomonas*, *Acinetobacter*, *Delftia*, *Pantoea*, *Stenotrophomonas*, *Rhizobium*, *Bacillus*, *Agrobacterium*, *Kosakonia*, and *Microbacterium* accounted for more than 1% of the microbial populations in healthy and diseased mulberry xylem. Overall, *Pseudomonas* and *Acinetobacter* were found to be the main constituent groups of the mulberry microbiome. This finding is supported by similar evidence reported by previous studies of Xu et al. (2019) and Ou et al. (2019), who reported that *Pseudomonas* was indeed an essential endophytic flora of mulberry. This evidence is further reinforced by reports of Suhaimi et al. (2017) and Kaushal et al. (2020) who also showed that *Pseudomonas* was an essential component of the banana bacterial flora. In our previous study, we found that *Pseudomonas* was one of the component of the endophytic flora in mulberry, but had no obvious control effect on the bacterial wilt of mulberry trees caused by *E. roggenkampii* strain KQ-01 (Yuan et al., 2023b). *Acinetobacter* was found to be an endophyte in mulberry and its proportion was significantly higher in mulberry varieties susceptible to bacterial wilt compared to the resistant varieties. In addition, the control rate of *Acinetobacter* against bacteria wilt caused by *E. roggenkampii* strain KQ-01 was higher than 80% (Yuan et al., 2023b). Seemingly, these results are contrasting and highlight that the precise roles played by *Pseudomonas* and *Acinetobacter* in plants need to be elucidated in future focused research.

Intriguingly, both metagenomic sequencing and culture-dependent methods employed in the present study showed that the proportion of *Pseudomonas* in the rhizosphere soil and xylem of healthy mulberry was higher compared to the diseased mulberry samples. Similarly, Suhaimi et al. (2017) showed that the abundance of *Pseudomonas* in the healthy banana samples was higher compared to the diseased samples. Using metagenomic sequencing, we found that the proportion of *Erwinia* bacteria in the rhizosphere soil and xylem of the diseased mulberry was higher compared to the healthy mulberry samples. However, this result was not supported by finding of the culture-dependent method. This finding is reinforced by evidence of our previous study in which a similar phenomenon was observed in mulberry samples (Yuan et al., 2023b).

In addition, the culture-dependent method revealed that the abundance of many opportunistic pathogens and drug-resistant bacteria was significantly higher in the xylem of the diseased samples compared to their healthy counterparts. Infections in humans have been reported mostly with opportunistic pathogens, including *Achromobacter* (Menetrey et al., 2021), *Acinetobacter* (Amorim and Nascimento, 2017)*, Brucella* (Roop et al., 2021), *Delftia* (Deb et al., 2020), *Escherichia* (Bhatt et al., 2019), *Herbaspirillum* (Bloise et al., 2021), *Klebsiella* (Rodríguez–Medina et al., 2019), *Ochrobactrum* (Bratschi et al., 2020), *Pantoea* (Cobo et al., 2021), *Ralstonia* (Ryan and Adley, 2014) and *Stenotrophomonas* (Menetrey et al., 2021). Moreover, *Acinetobacter* (Shin et al., 2020), *Escherichia* (Tang et al., 2022), *Klebsiella* (Dong et al., 2022), *Pantoea* (Yoshimura et al., 2022) and *Stenotrophomonas* (Ferreira et al., 2020) have been shown to have multidrug resistance.

In the present study, *Herbaspirillum*, *Klebsiella*, and *Ralstonia* were not isolated in the healthy mulberry xylem, indicating that these bacteria might have invaded after infection. Similar results were obtained by Hu et al. (2020), who found an increase in the relative abundance of *Ralstonia*, *Stenotrophomonas* and *Achromobacter* in the infected samples compared to the healthy samples. Although we suspect that the overuse of agricultural antibiotics and untreated farmyard manure exacerbates this situation, the precise underlying basis of this phenomenon remains to be explored. In addition, *Brenneria* was only isolated in the diseased mulberry samples but not in the healthy xylem. *Brenneria* has been reported to be a pathogen of woody plants that can cause cankers in plants including walnut (Poret–Peterson et al., 2019), oak (Denman et al., 2012), willow (Maes et al., 2009), alder (Maes et al., 2009) and poplar (Li et al., 2015). Currently, *Brenneria* is rarely reported in mulberry, and whether this pathogen is emerging as a new pathogen of mulberry still needs further investigation.

The culture-dependent method showed that many bacteria that have been reported to promote plant growth or control bacterial wilt were present in the mulberry samples. These included: *Agrobacterium* (Soares et al., 2020), *Microbacterium* (Singh and Singh, 2019), *Bacillus* (Im et al., 2020), *Lysinibacillus* (Lelapalli et al., 2021), *Oceanobacillus* (Alhindi and Albdaiwi, 2022), *Paenibacillus* (Abdallah et al., 2019), Enterobacter (Anand et al., 2021), *Kosakonia* (Brock et al., 2018), *Pseudomonas* (Zhuo et al., 2022) and *Streptomyces* (Olanrewaju and Babalola, 2019). In agreement with our finding, Hu et al. (2020) also reported similar results. They found that the relative abundance of *Pseudomonas*, *Bacillus*, and *Falsibacillus*, which are generally considered beneficial to plants, was significantly higher in the healthy mulberry samples compared to the diseased samples. This group of bacteria can be considered as a bank of beneficial microbial flora of mulberry. Interestingly, in the present study, the abundance of these bacteria was lower in the diseased samples compared to the healthy mulberry samples.

We further revealed that *Enterobacter* was the most widely distributed among the four types of pathogenic bacteria, accounting for 85.71%, followed by *Klebsiella* and *Pantoea*, which accounted for 34.28%. In contrast, *Ralstonia* accounted for the lowest (17.14%) proportion. This result indicated that *Enterobacter* might be the primary pathogen group causing bacterial wilt of mulberry, however, further focused research is needed to reinforce this evidence and gain more insights in this domain. Based on the *16S rDNA* sequence and its pathogenicity, *Ralstonia* was mainly clustered into two clades, the RSSC and *R. pickettii*. The pathogenicity of *Ralstonia* clustered in the same clade as the RSSC was greater than 45%, while clustered in the other clade, *R. pickettii* showed no pathogenicity. Meanwhile, *Enterobacter* was mainly clustered into the *ECC* and *E. lignolyticus*. A total of 73.91% of *Enterobacter* bacteria clustering in the *ECC* showed pathogenicity. *E. lignolyticus* clustered in one clade and showed no pathogenicity. *Klebsiella* was mainly clustered into two clades centered on *K. pneumoniae*, *K. quasipneumoniae*, *K. oxytoca*, and *K. michiganensis*, and both showed pathogenicity. *Pantoea* mainly clustered into two clades centered on *P. dispersa* and *P. anthophila* and did not show strong pathogenicity. However, *P. ananatis* strain LCFJ-001 (CP066803.1) which was discovered earlier (Yuan et al., 2023a) by our laboratory was shown to be pathogenic, with a pathogenicity rate of 38.33%. The RSSC, EC*C*, *K. pneumoniae*, *K. quasipneumoniae*, *K. oxytoca*, *K. michiganensis*, and *P. ananatis* were found to be the main components of the pathogenic bacteria of mulberry bacterial wilt.

During the RSSC infection, the Sol system can be regulated to produce an acylated homoserine lactone (AHL) quorum signaling factor, which is ubiquitous in various gram-negative bacteria, but it is poorly studied in the RSSC (Flavier et al., 1997). When AHL reaches a critical concentration, it diffuses into the cell to bind transcriptional regulators and activates other virulence regulators (Baltenneck et al., 2021). Density-dependent signaling systems centered on AHL are standard in gram-negative bacteria and have been reported in *Enterobacter* (Shastry et al., 2018), *Klebsiella* (Hosny and Fadel, 2021), and *Pantoea* (Jiang et al., 2015). It remains to be explored if there is a possibility that the RSSC can secrete enough AHL through the Sol regulation system to cooperate with other pathogenic bacteria and to infect together.

## Conclusion

The llumina HiSeq2500 sequencing and traditional culture medium approaches employed in the present study revealed that the bacterial diversity of healthy mulberry was higher compared to the diseased mulberry. The phyla Proteobacteria, Firmicutes and Actinobacteria constituted an important component of bacteria in the healthy and diseased mulberry. In addition, the abundance of many opportunistic pathogens and drug-resistant bacteria was significantly higher in the diseased samples compared to the healthy counterparts. It was found that the RSSC, ECC, *K. pneumoniae*, *K. quasipneumoniae*, *K. oxytoca*, *K. michiganensis*, and *P. ananatis* were the main components of the pathogenic bacteria of mulberry wilt. From these, the ECC was found to be the most widely distributed in the diseased samples. This study provides reference data for further focused research on the bacterial wilt of mulberry and other plants.

## Data availability statement

The datasets presented in this study can be found in online repositories. The names of the repository/repositories and accession number(s) can be found below: https://www.ncbi.nlm.nih.gov/, PRJNA911049; OP990608-OP990981;OP989957-OP990607.

## Author contributions

TY wrote the initial draft of manuscript, conceived experiment design, performed experiments, data analysis and implementation. IHQ participated in data analysis, interpretation of the results and revised and edited the draft. JHL, HY, PY, XZ, WL, and YQ collected materials, and assisted in the experiment and data analysis. JPL provided experimental platform and support, project supervision, and funding. All authors contributed to the article and approved the submitted version.
