# Supplementary material for: Analysis of changes in bacterial diversity in healthy and bacterial wilt mulberry samples using metagenomic sequencing and culture-dependent approaches
Source: Front Plant Sci. 2023 Aug 23;14:1206691. doi: 10.3389/fpls.2023.1206691 (PMC10481342; doi:10.3389/fpls.2023.1206691)
Supplement: Supplementary file 1 [file DataSheet_1.docx]

**Analysis of changes in bacterial diversity in healthy and bacterial wilt mulberry samples using metagenomic sequencing and culture-dependent approaches**

Ting Yuan^1†^, Izhar Hyder Qazi^1†^, Jinhao Li^1^, Peijia Yang^1^, Hongyu Yang^1^, Xueyin Zhang^1^, Weili Liu^1^, Jiping Liu^1*^

^1^South China Agriculture University, College of Animal Science, Regional Sericulture Training Center for Asia-Pacific, Wushan road, Guangzhou, Guangdong, 510642, China.

†These authors share first authorship

^*^Corresponding author: Jiping LIU (e-mail: [liujiping@scau.edu.cn](mailto:liujiping@scau.edu.cn))

**Supplementary tables and figures**

Supplementary table 1. Detailed description of samples used for metagenomic sequencing.

| Sample | Collection time | Location | Species | Field symptoms | Precipitation /mm | Temperature /℃ | Sampling site | Soil pH |
| --- | --- | --- | --- | --- | --- | --- | --- | --- |
| QKB04^**^ | 2018 | 109.24º, 24.65º | *M. atropurpurea* | Whole plant withered | 199.70 | 33.00 | Rhizosphere soil | 6.1~6.5 |
| QKB06^*^ | 2018 | 109.24º, 24.65º | *M. atropurpurea* | Normal | 199.70 | 33.00 | Rhizosphere soil | 6.9~7.5 |
| QKB08^**^ | 2018 | 109.35º, 25.15º | *M. atropurpurea* | Whole plant withered | 270.90 | 33.00 | Rhizosphere soil | 6.1~6.5 |
| QKB10^*^ | 2018 | 109.35º, 25.15º | *M. atropurpurea* | Normal | 270.90 | 33.00 | Rhizosphere soil | 6.9~7.5 |
| QKB03^**^ | 2018 | 109.24º, 24.65º | *M. atropurpurea* | Whole plant withered | 199.70 | 33.00 | Xylem | 6.1~6.5 |
| QKB05^*^ | 2018 | 109.24º, 24.65º | *M. atropurpurea* | Normal | 199.70 | 33.00 | Xylem | 6.9~7.5 |
| QKB07^**^ | 2018 | 109.35º, 25.15º | *M. atropurpurea* | Whole plant withered | 270.90 | 33.00 | Xylem | 6.1~6.5 |
| QKB09^*^ | 2018 | 109.35º, 25.15º | *M. atropurpurea* | Normal | 270.90 | 33.00 | Xylem | 6.9~7.5 |

QKB04: Liucheng (109.24º, 24.65º) diseased sample rhizosphere soil; QKB06: Liucheng (109.24º, 24.65º) healthy sample rhizosphere soil; QKB08: Rong'an (109.35º, 25.15º) diseased sample rhizosphere soil; QKB10: Rong'an (109.35º, 25.15º) healthy sample rhizosphere soil; QKB03: Liucheng (109.24º, 24.65º) diseased sample xylem; QKB05: Liucheng (109.24º, 24.65º) healthy sample xylem; QKB07: Rong'an (109.35º, 25.15º) diseased sample xylem; QKB09: Rong'an (109.35º, 25.15º) diseased sample xylem; "*": indicates the healthy group; "**" indicates the diseased group.

Supplementary table 2. The primers used in this study.

| Primer | Sequence |
| --- | --- |
| F | NNNNNNN[barcode]CTCCTACGGGAGGCAGCAG |
| R | CCVGGGTATCTAATCC |

N base can represent any one of A, T, G, or C. The barcode can be any 6-bp base sequence quickly resolved from other barcode sequences used in the same run.

Supplementary table 3. Description of bacterial wilt disease and healthy mulberry samples.

| N0. | Collection time | Location | Species | Precipitation /mm | Temperature /℃ | Sampling site | Quantity |
| --- | --- | --- | --- | --- | --- | --- | --- |
| MBWS1** | 2019.07.25 | 113.21º,22.71º | *M. atropurpurea* | 241.00 | 32.40 | Xylem | 1 |
| MBWS2** | 2019.07.25 | 112.64º,24.46º | *M. atropurpurea* | 203.90 | 34.00 | Xylem | 1 |
| MBWS3** | 2019.07.25 | 111.52º,22.58º | *M. atropurpurea* | 187.10 | 34.10 | Xylem | 1 |
| MBWS4** | 2020.05.20 | 108.57º,23.78º | *M. atropurpurea* | 262.00 | 29.80 | Xylem | 1 |
| MBWS5** | 2020.05.20 | 109.93º,23.98º | *M. atropurpurea* | 237.30 | 29.10 | Xylem | 1 |
| MBWS6** | 2020.05.20 | 109.35º,25.15º | *M. atropurpurea* | 320.30 | 28.20 | Xylem | 1 |
| MBWS7** | 2020.10.20 | 109.26º,24.52º | *M. atropurpurea* | 85.90 | 25.70 | Xylem | 1 |
| MBWS8** | 2020.10.20 | 110.43º,22.71º | *M. atropurpurea* | 57.90 | 29.20 | Xylem | 1 |
| MBWS9** | 2020.10.20 | 108.40º,24.21º | *M. atropurpurea* | 68.90 | 27.00 | Xylem | 1 |
| MBWS10** | 2020.11.15 | 108.12º,24.29º | *M. atropurpurea* | 49.00 | 22.40 | Xylem | 1 |
| MBWS11** | 2020.11.15 | 108.41º,24.31º | *M. atropurpurea* | 49.00 | 22.40 | Xylem | 1 |
| MBWS12** | 2020.11.15 | 109.79º,19.04º | *M. atropurpurea* | 180.30 | 25.20 | Xylem | 1 |
| MBWS13** | 2021.05.26 | 113.45º, 24.17º | *M. atropurpurea* | 307.10 | 29.30 | Xylem | 1 |
| MBWS14** | 2021.07.14 | 110.41º, 19.91º | *M. atropurpurea* | 214.70 | 33.50 | Xylem | 1 |
| MBWS15** | 2021.07.15 | 109.23º, 23.72º | *M. atropurpurea* | 214.40 | 33.20 | Xylem | 1 |
| MBWS16** | 2021.07.20 | 113.35º, 23.17º | *M. atropurpurea* | 240.00 | 33.70 | Xylem | 1 |
| MBWS17** | 2021.07.20 | 113.35º, 23.19º | *M. atropurpurea* | 240.00 | 33.70 | Xylem | 1 |
| MBWS18** | 2021.07.20 | 113.35º, 23.20º | *M. atropurpurea* | 240.00 | 33.70 | Xylem | 1 |
| MBWS19** | 2021.07.20 | 113.35º, 23.22º | *M. atropurpurea* | 240.00 | 33.70 | Xylem | 1 |
| MBWS20** | 2021.07.20 | 113.35º, 23.23º | *M. atropurpurea* | 240.00 | 33.70 | Xylem | 1 |
| MBWS21** | 2021.07.20 | 113.35º, 23.21º | *M. atropurpurea* | 240.00 | 33.70 | Xylem | 3 |
| MBWS22** | 2021.09.03 | 109.23º, 23.72º | *M. atropurpurea* | 82.60 | 31.70 | Xylem | 1 |
| MBWS23** | 2021.09.11 | 113.45º, 24.17º | *M. atropurpurea* | 110.00 | 31.80 | Xylem | 1 |
| MBWS24** | 2021.10.22 | 113.45º, 24.18º | *M. atropurpurea* | 43.60 | 28.50 | Xylem | 1 |
| MBWS25** | 2021.10.23 | 113.45º, 24.16º | *M. atropurpurea* | 43.60 | 28.50 | Xylem | 1 |
| MBWS26** | 2021.10.23 | 113.45º, 24.20º | *M. atropurpurea* | 43.60 | 28.50 | Xylem | 2 |
| MBWS27** | 2021.10.23 | 113.45º, 24.19º | *M. atropurpurea* | 43.60 | 28.50 | Xylem | 1 |
| MBWS28** | 2021.10.27 | 108.25º, 24.82º | *M. atropurpurea* | 67.40 | 26.90 | Xylem | 1 |
| MBWS29** | 2021.10.28 | 108.25º, 24.84º | *M. atropurpurea* | 67.40 | 26.90 | Xylem | 1 |
| MBWS30** | 2021.10.28 | 108.10º, 23.93º | *M. atropurpurea* | 72.90 | 27.90 | Xylem | 1 |
| MBWS31** | 2021.10.28 | 108.64º, 24.49º | *M. atropurpurea* | 64.60 | 27.30 | Xylem | 2 |
| MBWS32** | 2021.10.29 | 108.25º, 24.83º | *M. atropurpurea* | 67.40 | 26.90 | Xylem | 1 |
| MBWS33** | 2021.11.21 | 113.35º, 23.17º | *M. atropurpurea* | 38.40 | 25.30 | Xylem | 1 |
| MBWS34** | 2022.07.21 | 113.45º,24.17º | *M. atropurpurea* | 240.00 | 33.70 | Xylem | 2 |
| MBWS35** | 2022.07.21 | 108.25º, 24.82º | *M. atropurpurea* | 224.40 | 32.70 | Xylem | 1 |
| L* | 2022.07.21 | 113.35º, 23.17º | *M. atropurpurea* | 240.00 | 33.70 | Xylem | 20 |
| K* | 2022.07.21 | 113.35º, 23.17º | *M. atropurpurea* | 240.00 | 33.70 | Xylem | 20 |

"*" indicates the healthy group; "**" indicates the diseased group; MBWS: mulberry bacterial wilt sample. L: Mulberry cultivar Lun40; K: Mulberry cultivar Kangqing 10.

Supplementary table 4. Information of bacteria used to construct phylogenetic trees.

| Strain Name | Genus | Source | Classification by | GDMCC |
| --- | --- | --- | --- | --- |
| LKqk | *Ralstonia solanacearum* | Mulberry bacterial wilt | Specific primer detection | - |
| LZqk | *Ralstonia solanacearum* | Mulberry bacterial wilt | Specific primer detection | 1.1617 |
| XCqk | *Ralstonia solanacearum* | Mulberry bacterial wilt | Whole Genome Sequence | 1.1616 |
| XZqk | *Ralstonia solanacearum* | Mulberry bacterial wilt | Whole Genome Sequence | 1.1619 |
| YDqk | *Ralstonia solanacearum* | Mulberry bacterial wilt | Specific primer detection | 1.1620 |
| YLqk | *Ralstonia solanacearum* | Mulberry bacterial wilt | Whole Genome Sequence | 1.1615 |
| MBWS2.(6) | *Ralstonia* | MBWS2 | *16S rDNA* | - |
| MBWS6.(12) | *Ralstonia* | MBWS6 | *16S rDNA* | - |
| MBWS8.(25) | *Ralstonia* | MBWS8 | *16S rDNA* | - |
| MBWS12.(23) | *Ralstonia* | MBWS12 | *16S rDNA* | - |
| MBWS18.(1) | *Ralstonia* | MBWS18 | *16S rDNA* | - |
| MBWS35.(1) | *Ralstonia* | MBWS35 | *16S rDNA* | - |
| XCYG-001 | *Enterobacter asburiae* | Mulberry bacterial wilt | Whole Genome Sequence | 1.1600 |
| KQ-01 | *Enterobacter roggenkampii* | Mulberry bacterial wilt | Whole Genome Sequence | 1.1604 |
| MBWS1.(13) | *Enterobacter* | MBWS1 | *16S rDNA* | - |
| MBWS2.(10) | *Enterobacter* | MBWS2 | *16S rDNA* | - |
| MBWS3.(20) | *Enterobacter* | MBWS3 | *16S rDNA* | - |
| MBWS4.(19) | *Enterobacter* | MBWS4 | *16S rDNA* | - |
| MBWS5.(15) | *Enterobacter* | MBWS5 | *16S rDNA* | - |
| MBWS6.(10) | *Enterobacter* | MBWS6 | *16S rDNA* | - |
| MBWS7.(22) | *Enterobacter* | MBWS7 | *16S rDNA* | - |
| MBWS8.(4) | *Enterobacter* | MBWS8 | *16S rDNA* | - |
| MBWS9.(12) | *Enterobacter* | MBWS9 | *16S rDNA* | - |
| MBWS10.(15) | *Enterobacter* | MBWS10 | *16S rDNA* | - |
| MBWS12.(13) | *Enterobacter* | MBWS12 | *16S rDNA* | - |
| MBWS13.(7) | *Enterobacter* | MBWS13 | *16S rDNA* | - |
| MBWS14.(15) | *Enterobacter* | MBWS14 | *16S rDNA* | - |
| MBWS15.(7) | *Enterobacter* | MBWS15 | *16S rDNA* | - |
| MBWS17.(15) | *Enterobacter* | MBWS17 | *16S rDNA* | - |
| MBWS18.(19) | *Enterobacter* | MBWS18 | *16S rDNA* | - |
| MBWS19.(4) | *Enterobacter* | MBWS19 | *16S rDNA* | - |
| MBWS20.(4) | *Enterobacter* | MBWS20 | *16S rDNA* | - |
| MBWS22.(5) | *Enterobacter* | MBWS22 | *16S rDNA* | - |
| MBWS23.(1) | *Enterobacter* | MBWS23 | *16S rDNA* | - |
| MBWS24.(9) | *Enterobacter* | MBWS24 | *16S rDNA* | - |
| MBWS25.(16) | *Enterobacter* | MBWS25 | *16S rDNA* | - |
| MBWS26.(16) | *Enterobacter* | MBWS26 | *16S rDNA* | - |
| MBWS27.(4) | *Enterobacter* | MBWS27 | *16S rDNA* | - |
| MBWS28.(16) | *Enterobacter* | MBWS28 | *16S rDNA* | - |
| MBWS29.(3) | *Enterobacter* | MBWS29 | *16S rDNA* | - |
| MBWS30.(9) | *Enterobacter* | MBWS30 | *16S rDNA* | - |
| MBWS32.(5) | *Enterobacter* | MBWS32 | *16S rDNA* | - |
| MBWS33.(12) | *Enterobacter* | MBWS33 | *16S rDNA* | - |
| MBWS34.(11) | *Enterobacter* | MBWS34 | *16S rDNA* | - |
| AKKL-001 | *Klebsiella michiganensis* | Mulberry bacterial wilt | Whole Genome Sequence | 1.1602 |
| YDKL-002 | *Klebsiella quasipneumoniae* | Mulberry bacterial wilt | Whole Genome Sequence | 1.1603 |
| MBWS1.(12) | *Klebsiella* | MBWS1 | *16S rDNA* | - |
| MBWS4.(11) | *Klebsiella* | MBWS4 | *16S rDNA* | - |
| MBWS5.(17) | *Klebsiella* | MBWS5 | *16S rDNA* | - |
| MBWS6.(8) | *Klebsiella* | MBWS6 | *16S rDNA* | - |
| MBWS9.(7) | *Klebsiella* | MBWS9 | *16S rDNA* | - |
| MBWS10.(16) | *Klebsiella* | MBWS10 | *16S rDNA* | - |
| MBWS11.(8) | *Klebsiella* | MBWS11 | *16S rDNA* | - |
| MBWS13.(8) | *Klebsiella* | MBWS13 | *16S rDNA* | - |
| MBWS14.(14) | *Klebsiella* | MBWS14 | *16S rDNA* | - |
| MBWS28.(6) | *Klebsiella* | MBWS28 | *16S rDNA* | - |
| MBWS31.(7) | *Klebsiella* | MBWS31 | *16S rDNA* | - |
| MBWS32.(30) | *Klebsiella* | MBWS32 | *16S rDNA* | - |
| LCFJ-001 | *Pantoea ananatis* | Mulberry bacterial wilt | Whole Genome Sequence | 1.1601 |
| MBWS1.(11) | *Pantoea* | MBWS1 | *16S rDNA* | - |
| MBWS3.(2) | *Pantoea* | MBWS3 | *16S rDNA* | - |
| MBWS4.(6) | *Pantoea* | MBWS4 | *16S rDNA* | - |
| MBWS7.(13) | *Pantoea* | MBWS7 | *16S rDNA* | - |
| MBWS11.(2) | *Pantoea* | MBWS11 | *16S rDNA* | - |
| MBWS13.(17) | *Pantoea* | MBWS13 | *16S rDNA* | - |
| MBWS16.(13) | *Pantoea* | MBWS16 | *16S rDNA* | - |
| MBWS17.(3) | *Pantoea* | MBWS17 | *16S rDNA* | - |
| MBWS18.(5) | *Pantoea* | MBWS18 | *16S rDNA* | - |
| MBWS19.(6) | *Pantoea* | MBWS19 | *16S rDNA* | - |
| MBWS24.(16) | *Pantoea* | MBWS24 | *16S rDNA* | - |
| MBWS32.(20) | *Pantoea* | MBWS32 | *16S rDNA* | - |

GDMCC: Guangdong Microbial Culture Collection Center; “-”: Preserved in our laboratory

Supplementary table 5. Cumulative list of cultivable xylem bacteria and their taxonomic information in healthy mulberry.

| Phyla | Classes | Orders | Families | Genera |
| --- | --- | --- | --- | --- |
| Actinobacteria | Actinomycetia | Micrococcales | Cellulomonadaceae | *Cellulomonas* (2) |
|  |  |  | Microbacteriaceae | *Agrococcus* (4) |
|  |  |  |  | *Agromyces* (1) |
|  |  |  |  | *Curtobacteriu* (2) |
|  |  |  |  | *Microbacterium* (7) |
|  |  |  | Micrococcaceae | *Arthrobacter* (1) |
|  |  |  |  | *Glutamicibacter* (3) |
|  |  |  |  | *Micrococcus* (2) |
|  |  |  | Promicromonosporaceae | *Cellulosimicrobium* (2) |
|  |  |  |  | *Isoptericola* (2) |
|  |  | Propionibacteriales | Nocardioidaceae | *Nocardioides* (1) |
|  |  | Corynebacteriales | Nocardiaceae | *Prescottella* (2) |
|  |  | Streptomycetales | Streptomycetaceae | *Streptomyces* (6) |
| Bacteroidota | Flavobacteriia | Flavobacteriales | Weeksellaceae | *Chryseobacterium* (2) |
| Firmicutes | Bacilli | Bacillales | Bacillaceae | *Bacillus* (31) |
|  |  |  |  | *Cytobacillus* (1) |
|  |  |  |  | *Exiguobacterium* (2) |
|  |  |  |  | *Fictibacillus* (1) |
|  |  |  |  | *Lysinibacillus* (5) |
|  |  |  |  | *Oceanobacillus* (4) |
|  |  |  |  | *Peribacillus* (3) |
|  |  |  |  | *Priestia* (1) |
|  |  |  |  | *Psychrobacillus* (3) |
|  |  |  |  | *Solibacillus* (1) |
|  |  |  |  | *Siminovitchia* (1) |
|  |  |  | Paenibacillaceae | *Paenibacillus* (11) |
|  |  |  | Planococcaceae | *Bhargavaea* (2) |
|  |  |  |  | *Sporosarcina* (1) |
|  |  |  | Staphylococcaceae | *Macrococcus* (2) |
|  |  |  |  | *Staphylococcus* (7) |
| Proteobacteria | Alphaproteobacteria | Burkholderiales | Alcaligenaceae | *Achromobacter* (2) |
|  |  | Caulobacterales | Caulobacteraceae | *Brevundimonas* (1) |
|  |  | Hyphomicrobiales | Brucellaceae | *Brucella* (1) |
|  |  |  |  | *Ochrobactrum* (1) |
|  |  |  | Phyllobacteriaceae | *Mesorhizobium* (3) |
|  |  |  | Rhizobiaceae | *Ensifer* (2) |
|  |  |  |  | *Agrobacterium* (9) |
|  |  |  |  | *Rhizobium* (9) |
|  | Betaproteobacteria | Sphingomonadales | Sphingomonadaceae | *Sphingobium* (1) |
|  |  | Burkholderiales | Burkholderiaceae | *Cupriavidus* (1) |
|  |  | Neisseriales | Neisseriaceae | *Neisseria* (1) |
|  |  | Burkholderiales | Alcaligenaceae | *Alcaligenes* (3) |
|  |  |  | Comamonadaceae | *Xenophilus* (1) |
|  |  |  |  | *Comamonas* (2) |
|  |  |  |  | *Delftia* (9) |
|  | Gammaproteobacteria | Aeromonadales | Aeromonadaceae | *Aeromonas* (1) |
|  |  | Enterobacterales | Enterobacteriaceae | *Atlantibacter* (6) |
|  |  |  |  | *Enterobacter* (77) |
|  |  |  |  | *Escherichia* (2) |
|  |  |  |  | *Kluyvera* (1) |
|  |  |  |  | *Kosakonia* (9) |
|  |  |  |  | *Salmonella* (2) |
|  |  |  | Erwiniaceae | *Pantoea* (13) |
|  |  | Moraxellales | Moraxellaceae | *Moraxella* (1) |
|  |  |  |  | *Acinetobacter* (23) |
|  |  | Pseudomonadales | Pseudomonadaceae | *Pseudomonas* (80) |
|  |  | Xanthomonadales | Xanthomonadaceae | *Xanthomonas* (3) |
|  |  |  |  | *Stenotrophomonas* (12) |

Supplementary table 6. Cumulative list of cultivable xylem bacteria and their taxonomic information in diseased mulberries.

| Phyla | Classes | Orders | Families | Genera |
| --- | --- | --- | --- | --- |
| Actinobacteria | Actinomycetia | Corynebacteriales | Nocardiaceae | *Rhodococcus* (1) |
|  |  | Micrococcales | Dermabacteraceae | *Brachybacterium* (1) |
|  |  |  | Microbacteriaceae | *Curtobacteriu* (4) |
|  |  |  |  | *Microbacterium* (8) |
|  |  |  | Micrococcaceae | *Arthrobacter* (3) |
|  |  |  |  | *Glutamicibacter* (1) |
| Bacteroidota | Chitinophagia | Chitinophagales | Chitinophagaceae | *Chitinophaga* (5) |
|  |  |  |  | *Pseudobacter* (1) |
|  | Cytophagia | Cytophagales | Spirosomaceae | *Dyadobacter* (4) |
|  | Flavobacteriia | Flavobacteriales | Flavobacteriaceae | *Flavobacterium* (2) |
|  |  |  | Weeksellaceae | *Chryseobacterium* (6) |
|  | Sphingobacteriia | Sphingobacteriales | Sphingobacteriaceae | *Olivibacter* (2) |
|  |  |  |  | *Sphingobacterium* (4) |
| Firmicutes | Bacilli | Bacillales | Bacillaceae | *Bacillus* (16) |
|  |  |  |  | *Lederbergia* (2) |
|  |  |  |  | *Lysinibacillus* (4) |
|  |  |  |  | *Priestia* (2) |
|  |  |  |  | *Rossellomorea* (1) |
|  |  |  |  | *Terribacillus* (1) |
|  |  |  |  | *Oceanobacillus* (1) |
|  |  |  | Staphylococcaceae | *Mammaliicoccus* (1) |
|  |  |  |  | *Staphylococcus* (1) |
|  |  | Lactobacillales | Streptococcaceae | *Lactococcus* (2) |
| Proteobacteria | Alphaproteobacteria | Caulobacterales | Caulobacteraceae | *Caulobacter* (2) |
|  |  | Hyphomicrobiales | Brucellaceae | *Brucella* (14) |
|  |  |  |  | *Ochrobactrum* (9) |
|  |  |  | Phyllobacteriaceae | *Mesorhizobium* (1) |
|  |  |  | Rhizobiaceae | *Agrobacterium* (10) |
|  |  |  |  | *Rhizobium* (23) |
|  |  |  |  | *Shinella* (2) |
|  |  |  | Xanthobacteraceae | *Starkeya* (2) |
|  |  | Rhodospirillales | Acetobacteraceae | *Acetobacter* (1) |
|  |  | Sphingomonadales | Sphingomonadaceae | *Sphingobium* (1) |
|  |  |  |  | *Sphingopyxis* (1) |
|  | Betaproteobacteria | Burkholderiales | Alcaligenaceae | *Achromobacter* (34) |
|  |  |  |  | *Alcaligenes* (5) |
|  |  |  |  | *Bordetella* (2) |
|  |  |  |  | *Castellaniella* (1) |
|  |  |  |  | *Pigmentiphaga* (1) |
|  |  |  | Burkholderiaceae | *Burkholderia* (1) |
|  |  |  |  | *Cupriavidus* (6) |
|  |  |  |  | *Pandoraea* (1) |
|  |  |  |  | *Ralstonia* (6) |
|  |  |  | Comamonadaceae | *Comamonas* (3) |
|  |  |  |  | *Delftia* (34) |
|  |  |  |  | *Diaphorobacter* (5) |
|  |  |  | Oxalobacteraceae | *Herbaspirillum* (7) |
|  |  |  | Sphaerotilaceae | *Mitsuaria* (2) |
|  | Gammaproteobacteria | Enterobacterales | Enterobacteriaceae | *Atlantibacter* (1) |
|  |  |  |  | *Citrobacter* (2) |
|  |  |  |  | *Cronobacter* (2) |
|  |  |  |  | *Enterobacter* (117) |
|  |  |  |  | *Escherichia* (8) |
|  |  |  |  | *Klebsiella* (18) |
|  |  |  |  | *Kosakonia* (9) |
|  |  |  |  | *Leclercia* (2) |
|  |  |  |  | *Lelliottia* (2) |
|  |  |  |  | *Pseudocitrobacter* (1) |
|  |  |  |  | *Raoultella* (2) |
|  |  |  |  | *Siccibacter* (1) |
|  |  |  | Erwiniaceae | *Erwinia* (1) |
|  |  |  |  | *Pantoea* (33) |
|  |  |  |  | *Rosenbergiella* (4) |
|  |  |  | Pectobacteriaceae | *Brenneria* (13) |
|  |  |  | Yersiniaceae | *Serratia* (6) |
|  |  | Moraxellales | Moraxellaceae | *Acinetobacter* (56) |
|  |  | Pseudomonadales | Pseudomonadaceae | *Pseudomonas* (107) |
|  |  | Xanthomonadales | Xanthomonadaceae | *Stenotrophomonas* (29) |
|  |  |  |  | *Xanthomonas* (3) |


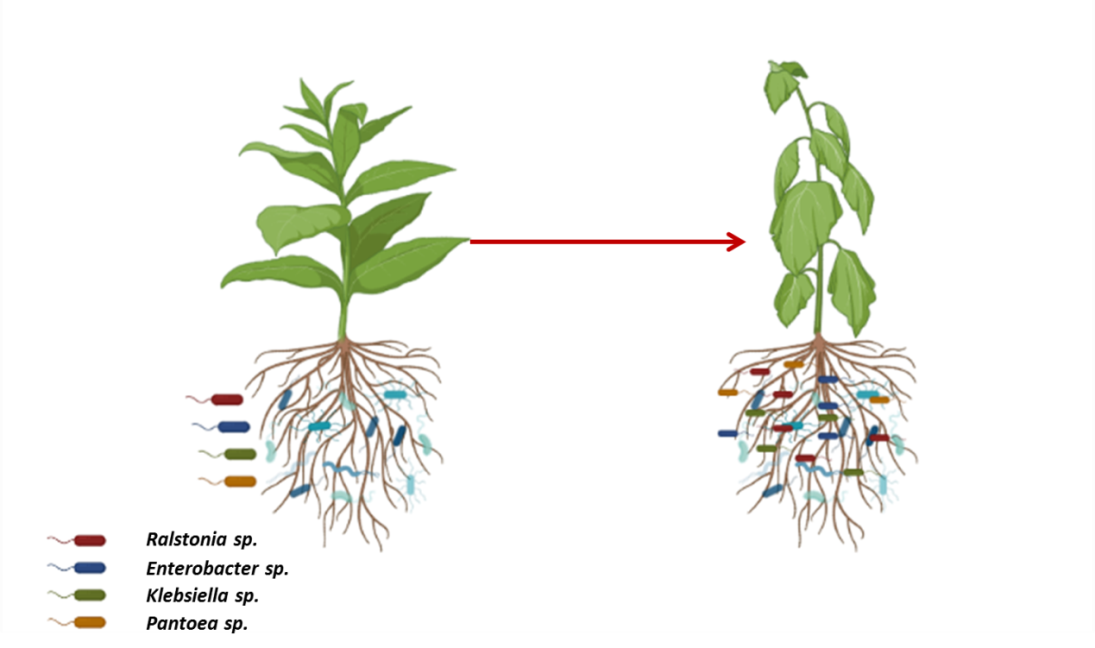


Supplementary figure 1. Pattern diagram of bacterial wilt of mulberry

Supplementary figure 2. Symptoms of mulberry wilt bacterial wilt in the field and symptoms of roots and branches during the onset period. (A) and (B) are the field symptoms of mulberry wilt (*M. atropurpurea*) in Aikou Village, Rong’an County, Guangxi, China (109.35º, 25.15º); (C), (D), and (E) are Fengshan, Liucheng (109.24º, 24.65º) field symptoms of the same mulberry field in the demonstration field (*M. atropurpurea*); (F) Field symptoms of mulberry in the Fengshan demonstration field in Liucheng (*M. atropurpurea*); (G) Mulberry in the Fengshan demonstration garden in Liucheng (*M. atropurpurea*); (H) Symptoms of mulberry wilt root disease (*M. atropurpurea*); (I) Characters of mulberry wilt branch xylem (left) and normal branch xylem (right).


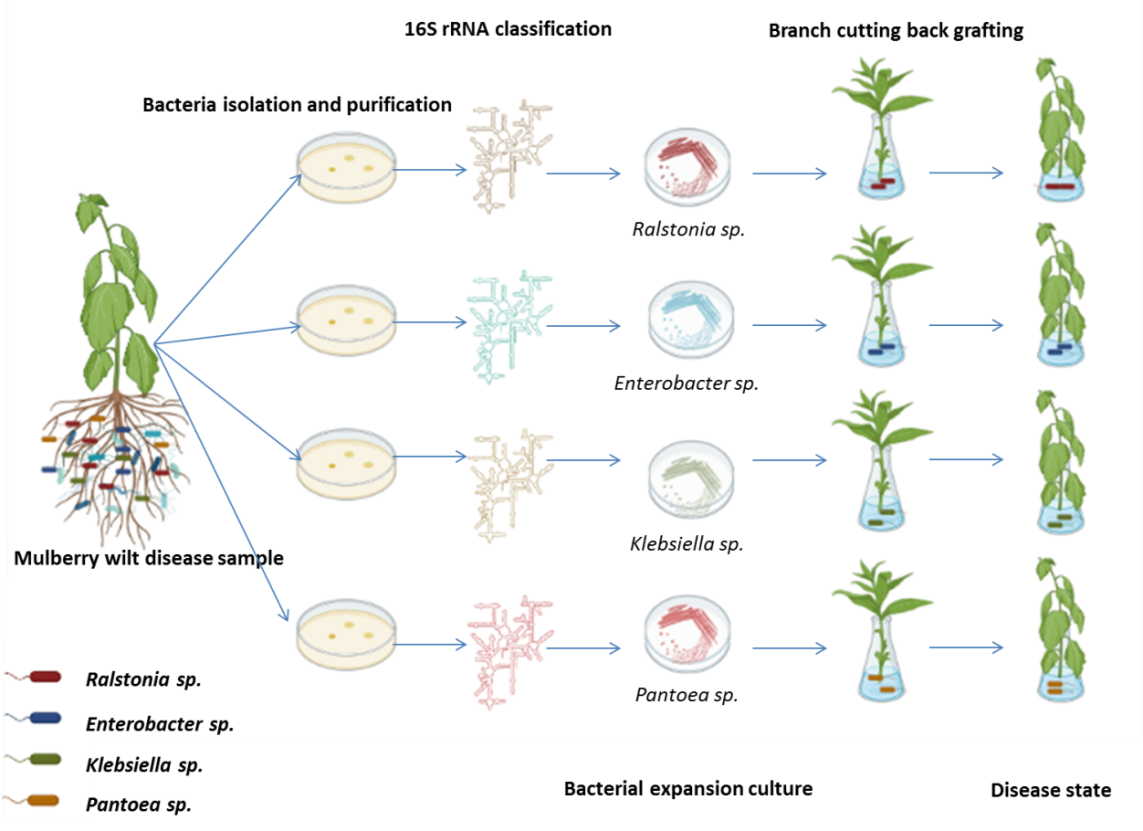


Attached figure 3. Schematic of experimental steps used in culture-dependent approach for mulberry wilt bacterial wilt samples.
